# Phantosmia and Benzodiazepine Withdrawal: A Case Report

**DOI:** 10.7759/cureus.92116

**Published:** 2025-09-12

**Authors:** Miki Kiyokawa, Saige R Fong, Micaiah C Cape

**Affiliations:** 1 Department of Psychiatry, University of Hawaii, John A. Burns School of Medicine (JABSOM), Honolulu, USA

**Keywords:** benzodiazepine withdrawal, chronic benzodiazepine use, olfactory hallucinations, phantom smell, phantosmia, sedative withdrawal

## Abstract

Olfactory hallucinations, known as phantosmia, involve the perception of smell without the presence of existing chemical stimuli and are usually described as unpleasant. Other than a single brief mention as one of the symptoms of benzodiazepine withdrawal, olfactory hallucinations are not specifically listed as a symptom of benzodiazepine withdrawal in the common literature.

We report the case of an older female with no previous history of psychosis, taking clonazepam for anxiety for five years. She was instructed by her outpatient provider to stop clonazepam before her elective surgery. Due to fear of mixing oxycodone prescribed for postoperative pain, the patient did not resume taking clonazepam during the hospitalization after surgery. No education was provided about benzodiazepine withdrawal or its symptoms. After discharge, she continued to hold clonazepam and started to experience a series of symptoms, such as insomnia, increased sensitivity to smell, and hearing music in her ears. On the day of admission, 14 days after her last clonazepam, she presented with the perception of an unpleasant odor, which she described as “bad” and “evil.” Clonazepam was restarted, and all symptoms promptly resolved.

To the best of our knowledge, no such case has been reported before. This case illustrates a rare presentation of olfactory hallucinations most likely precipitated by benzodiazepine withdrawal that was completely reversed by restarting the medication. In addition, it emphasizes the importance of providers and patient education regarding safe benzodiazepine discontinuation and raising awareness that olfactory hallucinations may present as a symptom of benzodiazepine withdrawal.

## Introduction

Olfactory hallucination, also known as phantom smells, or phantosmia, is defined as a person detecting a smell without any existing chemical stimuli [1,2]. It is considered a qualitative olfactory dysfunction [1]. These phantom odors are usually unpleasant, with patients often describing smells with terms such as “foul,” “rotten,” or “chemical” [1]. Phantosmia has a higher frequency among young adults (under 40 years old), women, individuals of lower socioeconomic status, and those with poor health or dry mouth symptoms [1,2]. Phantosmia has also been observed after upper respiratory infections (including sinonasal disease), head trauma, migraines, epilepsy, neurodegenerative disease, and radiation therapy. It may also occur with psychological factors such as bipolar disorder, schizophrenia, and depression [1,2]. Psychoactive substances or medications may also trigger olfactory hallucinations. In general, olfactory hallucinations appear to be rare, with about 80% of cases lasting less than a few minutes [2]. Smoking or drinking alcohol is not known to increase prevalence. Causations proposed include abnormal signaling or inhibition from primary olfactory neurons or triggering of central processes by peripheral olfactory or trigeminal nerves [3]. To date, the cause of phantosmia is not entirely understood [1].

Benzodiazepines (i.e., lorazepam and clonazepam) are a widely prescribed class of drugs in different populations and care settings [4]. Benzodiazepine use is rising globally, including in the U.S. An article published in the Journal of the American Medical Association (2019) reported that U.S. benzodiazepine visits increased from an estimated 27.6 million in 2003 to 62.6 million in 2015, doubling among adults ≥ 45 to 64 years old [5]. Benzodiazepines generally increase the risk of falls, confusion, motor vehicle accidents, and memory impairment, prompting their avoidance in older adults per the Beers criteria [6]. The rise in benzodiazepine use is alarming, with increases in related deaths, emergency room visits, and complications from ingestion with other substances like alcohol and opioids [5].

Benzodiazepines act as allosteric modulators in the central nervous system by increasing the affinity of the major inhibitory neurotransmitter gamma-aminobutyric acid (GABA) for its GABA-A binding site, leading to increased chloride ion influx and neuronal hyperpolarization [7]. Half-lives vary depending on the specific formulation. Desired effects include reduction of anxiety, induction of sleep, muscle relaxation, and prevention of epileptic seizures; adverse effects include drowsiness, mental slowing, and memory disturbance [7]. Benzodiazepines are considered relatively safe for short-term use (two to four weeks). Dependence, however, can occur after as short as one month of treatment in some patients [7].

Long-term use of benzodiazepines has the potential to cause severe withdrawal symptoms upon discontinuation. With long-acting benzodiazepines, such as clonazepam (elimination half-life of 18 to 50 hours) [8], withdrawal symptoms occur three to seven days after discontinuation, and symptoms may persist for months [9,10]. Withdrawal symptoms reflect central nervous system hyperexcitability; common symptoms include tremors, anxiety, insomnia, hypersensitivity to stimuli, seizures, delirium, and hallucinations (visual is most common) [9,11,12]. Withdrawal symptoms may also vary with age: younger individuals are more likely to experience agitation, anxiety, insomnia, and generalized seizures, while older adults (aged >50 years) may present with confusion, hallucinations, and catatonia [9]. While visual and auditory hallucinations associated with benzodiazepine withdrawal are documented in the literature, to the best of our knowledge, our PubMed/Google search found no case reports of olfactory hallucinations linked to benzodiazepine withdrawal other than a brief mention in the Ashton manual [9-13]. 

We report a case of phantosmia in a patient whose chronic clonazepam prescription was discontinued for elective surgery. Due to fear of mixing opioid pain medication post-procedure, she continued to withhold taking clonazepam. Phantosmia and other symptoms resolved completely after clonazepam was restarted. The patient consented to have her case presented as a report, and all necessary steps were taken as instructed by our institutional review board case report protocol. As this was a case report of a single patient, it was exempt from review by our research ethics board.

## Case presentation

A healthy female in her mid-60s had been taking clonazepam 1 mg twice daily (sometimes, an additional 1 mg at night) for anxiety for the past five years. She presented to the emergency department complaining of unpleasant odor, anorexia, and weakness. She had no history of hallucination, psychosis, or psychiatric hospitalization. Medical, family, and psychosocial history were noncontributory.

She followed the instructions given by her outpatient provider and discontinued clonazepam 14 days prior to admission (PTA) in preparation for elective surgery. Eight days PTA, she successfully underwent the procedure, and clonazepam was restarted during the hospitalization. She had no complaints or symptoms of benzodiazepine withdrawal at that time. Due to fear of mixing oxycodone prescribed for postoperative pain, the patient refused clonazepam throughout her hospital course. She reported that her providers neither encouraged clonazepam use nor discussed withdrawal risks with her when she declined the medication during hospitalization. She continued to withhold clonazepam after discharge but used oxycodone for pain.

Four days PTA (11 days from her last clonazepam), the patient started to hear music in her ears. The music she was hearing was a genre she was familiar with. This was accompanied by anxiety, insomnia, and increased sensitivity to various smells, which led to poor oral intake. For a brief moment, she felt “she was going to die.” She thought her symptoms might be due to oxycodone, so she stopped taking it but did not resume her clonazepam. One day prior to admission, her sensitivity to aroma increased, and her anorexia persisted. She also started to have tremors and continued to hear music in her ears. On the day of admission, the patient noted photosensitivity and an unpleasant odor that she described as “bad” and “evil,” in addition to tremors, insomnia, and music in her ears. She was unable to eat because of the continuous unpleasant, malodorous smell, which she further described as "rotten." She reported that nothing exacerbated or relieved the unpleasant smell.

In the emergency department, she was noted to be cooperative with no obvious agitation. Her mentation was noted to be appropriate, and she provided a history, which was concurred by the family at the bedside. Her physical exam was significant for slight tremors, dry mucous membranes, tachycardia, and a non-focal neurological exam. She appeared anxious, and the lab showed hypokalemia. All other labs obtained were unremarkable. The emergency room provider and psychiatrist ruled out common etiologies of phantosmia, including infection and neurological disorders. She was admitted for benzodiazepine withdrawal in addition to dehydration and hypokalemia from poor oral intake. After fluid and electrolyte replacement, the patient’s tachycardia and hypokalemia improved. Her clonazepam was restarted at 1 mg twice daily. The patient fell asleep shortly after the first dose of clonazepam. The next morning, the patient reported that she slept well, and when she woke up, she realized she no longer had any symptoms, including photosensitivity, music in her ears, and olfactory hallucinations. The patient was able to intake a full meal, and she was discharged in an improved state. Please see Table 1 for the timeline and symptom development.

**Table 1:** Timeline of discontinuation of clonazepam and symptoms

Day without clonazepam	Day prior to admission (PTA)	Major events	Symptoms experienced
Day 1 - Day 6	14 days PTA to 9 days PTA	Clonazepam was stopped in preparation for elective surgery	No symptoms
Day 7	8 days PTA	Admitted to the hospital for elective surgery, and oxycodone was started for postoperative pain. Clonazepam was ordered, but the patient refused.	No symptoms
Day 8	7 days PTA	Took oxycodone but refused clonazepam	No symptoms
Day 9	6 days PTA	Took oxycodone but refused clonazepam	No symptoms
Day 10	5 days PTA	Took oxycodone but refused clonazepam. The patient was discharged home later in the day and continued to stay away from clonazepam.	No symptoms
Day 11	4 days PTA	Took oxycodone but discontinued at the end of the day, thinking her symptoms were caused by oxycodone	Reported anxiety, insomnia, music in her ears, and increased sensitivity to smell, which led to decreased oral intake
Day 12	3 days PTA	No oxycodone taken	Reported that the above symptoms persisted
Day 13	2 days PTA	No oxycodone taken	Reported that the above symptoms persisted
Day 14	1 day PTA	No oxycodone taken	Tremors in addition to the above symptoms, with worsening of hypersensitivity to smell
Day 15	Day of admission	No oxycodone taken. The patient was admitted, and clonazepam was restarted.	Came to the hospital complaining of photosensitivity and unable to eat from the unpleasant odor described as "bad" and "evil", which led to weakness. She appeared anxious and reported tremors, insomnia, and music in her ears.

## Discussion

Olfactory hallucinations are predominantly characterized by the perception of unpleasant odors in the absence of external stimuli [1]. Other than the brief mention in the Ashton manual, our PubMed/Google search identified various benzodiazepine withdrawal symptoms, such as anxiety, insomnia, mood disturbances, and various hallucinations, but not phantosmia [9-13]. Withdrawal from other sedatives, such as barbiturates and GABA-B receptor agonists (including baclofen, gamma-hydroxybutyrate, and phenibut), may manifest with visual, auditory, and/or tactile hallucinations; however, to the best of our knowledge, no cases specifically mention phantosmia as a withdrawal symptom [9,14-16]. Interestingly, a recent case report suggests that olfactory hallucinations can occur as a unique presentation of antidepressant discontinuation syndrome [17]. 

The patient initially started to manifest symptoms of withdrawal on the 11th day after she discontinued clonazepam. This interval of time is slightly later than expected, as the onset of longer-acting benzodiazepine withdrawal is within three to seven days [9]. This observation may be explained by the patient's age (the elderly have slower metabolism [6]; hence, the appearance of withdrawal symptoms may be delayed) and her taking oxycodone postoperatively for acute pain. Although oxycodone and benzodiazepines target different receptors, studies have shown that opioids can help to reduce anxiety in both animals and humans [18,19]. In this case, oxycodone was used for postoperative pain, which may have masked the initial minor benzodiazepine withdrawal symptoms such as anxiety. Her series of symptoms, including olfactory hallucinations, was most likely caused by clonazepam withdrawal from history. All symptoms reversed after restarting outpatient clonazepam. 

Benzodiazepine withdrawal may lead to severe complications, but these can be prevented or at least minimized through implementation of safe practices, such as the Joint Clinical Practice Guideline on Benzodiazepine Tapering [20]. In this case, multidisciplinary members of the healthcare team could have communicated information about benzodiazepine discontinuation, withdrawal symptoms, and the importance of resuming clonazepam as directed to avoid withdrawal symptoms at multiple timepoints during post-procedure hospitalization. In addition to a rare description of olfactory hallucinations most likely associated with benzodiazepine withdrawal, this case raises the importance of educating providers on safe prescribing practices and informing patients about symptoms associated with benzodiazepine discontinuation. 

## Conclusions

This case presents a rare description of olfactory hallucinations most likely associated with benzodiazepine withdrawal, contributing valuable insight to a topic that is infrequently addressed in the existing literature. Due to fear of mixing with oxycodone, the patient did not resume clonazepam as directed. She developed a series of uncomfortable withdrawal symptoms, which resolved when the medication was restarted. The patient’s hospitalization was most likely preventable if she was educated about the importance of resuming her clonazepam. This case also raises the importance of educating providers and patients about safe benzodiazepine use and associated benzodiazepine-related symptoms. 
